# Essays on the Puerperal Fever and Other Diseases Peculiar to Women

**Published:** 1850-07

**Authors:** 


Art. X.—*Essays on the Puerperal Fever and other Diseases peculiar to Women. Selected from the Writings of British Authors previous to the close of the Eighteenth Century.* Edited by Fleetwood Churchill, M. D., M. R. J. A., Hon. Fellow of the College of Physicians of Ireland; Corresponding Member of the American National Institnte, &c. Philadelphia: Lea & Blanchard. 1850.

Many of our readers are probably aware of the fact, that a society in England, under the appropriate name of the Sydenham Society, is engaged in the republication of all the valuable old medical literature of England, and, in this way, is rescuing all that is of interest from that species of oblivion that takes possession of works, that are said to be, in bookseller's phrase, "out of print." The work before us is one of the results of the happy idea upon which the "Sydenham Society" was formed, and that society showed an admirable degree of discrimination in selecting Dr. Churchill for editing this work.

The field proved to be more barren of fruit than Dr. Churchill supposed when he undertook the work presribed for him. He says: "On careful examination, I found a considerable number of interesting *cases* recorded; but extremely few *papers* of a sufficient length and importance to justify their republication. Until the latter half of the last century there was no book on "Diseases of Women" of any scientific pretension, and we cannot, therefore, wonder that monographs were scarce. Since that time the subject has obtained due attention, and now it is not behind any other department, in the importance attached to it, the value of the works illustrating it, and the scientific character of the practitioners pursuing it."

The greater portion of this work is occupied with papers on puerperal fever, a disease that rarely assumes any approach to an epidemic form in the West or South. But the disease is met with often enough to require practitioners to understand it thoroughly.

The work of Dr. Churchill opens with an excellent history of epidemics of puerperal fever. There is no doubt but that Hippocrates and Avicenna were well acquainted with the disease, and they both referred it to an obstruction of the lochia. The first undoubted epidemic of puerperal fever on record, our author thinks was the one that prevailed in Paris, during the winter of 1766. It was more fatal among hospital patients, than among those who were delivered at home. In 1750, an epidemic of puerperal fever appeared at Lyons, and at Paris. In 1760, eleven years after the establishment of lying-in-hospitals in England, the first recorded account of epidemic puerperal fever appeared. In 1767, the disease appeared in Dublin hospital, about ten years after that institution was first opened for patients. Puerperal fever appeared three times between 1774 and 1788.

In the Westminster hospital, between November, 1769, and May, 1770, out of sixty-three delivered, nineteen had puerperal fever, and fourteen died.

Dr. Wm. Hunter was in the habit of informing his pupils that, of thirty-two patients attacked by the disease in two months, only one recovered; that, treat them in what manner you please, at least three out of four will die.

In 1770, a puerperal epidemic appeared in the hospital of St. Mark, Vienna. In 1773, the disease appeared in the lying-in ward of the Royal Infirmary, of Edinburgh. In 1774–'5, puerperal fever, prevailed extensively in Paris. The disease prevailed in Aberdeen, Scotland, in 1760–'61. From 1765 to 1775, puerperal fever appears to have prevailed in Derbyshire and the adjacent counties. In 1785, a fatal puerperal epidemic prevailed in the Vienna lying-in hospital. In 1787, the disease was prevalent and fatal in London. In 1789, the epidemic renewed its ravages in Aberdeen.

Mr. Key mentions that an epidemic of puerperal fever commenced in Yorkshire in 1808, and at Leeds in 1809, continuing in the latter town until Christmas, 1812. "*It was coincident with an epidemic of erysipelas.*" This is the first reference we have found to erysipelas, in this history.

Dr. Collins says that puerperal fever was epidemic in the lying-in hospital in Dublin, in the year 1803, and 1810–'13–'18–'19–'20–'23–'26–'28–'29.

Dr. Douglass, in a sketch published in 1810, refers to erysipelatous inflammation in connection with puerperal fever.

This meager sketch brings us down to a period, when the literature of medicine began to take a great deal of notice of epidemic puerperal fever. The publications of Denman, Gordon, Gooch, Armstrong, and Mackintosh, made the subject of puerperal fever familiar to the profession.

Dr. Churchill gives a tabular statement, containing the localities of epidemic puerperal fever, from 1664 to 1846, inclusive, and of the local disease in each visitation from 1664 to 1836, inclusive.

The following facts are mentioned by Dr. Churchill as the result of his investigations: 1. There appears to be a special connection between epidemics of puerperal fever, and lying-in hospitals.

2. An almost universal connection between puerperal fever and local disease.

3. Puerperal fever previals most during the winter or spring months, and in moist and cold weather, or with alternations of cold and warm moist weather.

4. There are three diseases that appear to prevail concurrently with epidemic puerperal fever! They are bowel complaints, (or gastroenteritis,) typhus fever, and erysipelas. Dr. Churchill thinks, we believe, very correctly, that puerperal fever and erysipelas are essentially the same disease. Upon this subject we think there is but little room for doubt.

On the question of contagiousness there is a great diversity of opinion, a pretty conclusive proof, in our judgment, of the non-contagiousness of the disease. There is no difference of opinion among medical men on the contagion of small pox. Such minds as are opposed upon the contagion and non-contagion of puerperal fever are not generally found opposed to each other, on the facts that occur in an open field of observation. We cannot possibly conceive of the existence of such a thing as a disease non-contagious in one set of circumstances, and contagious in another set. If a disease is contagious, contagion is an essential element of its nature, and we know of no power that can impart to it, what does not belong to it. Those who contend for the contagiousness of puerperal fever, must contend also for a sex in contagion. A female may give a man the small pox, but we have never heard of any one who imagined it possible for a female to impart puerperal fever to one of the other sex. We have met with but few American physicians, who think puerperal fever contagious; we could as easily believe chills and fever communicable as to believe in the contagion of either sporadic or epidemic puerperal fever. Medical men are somewhat prone to believe that accidental coincidences bear the relation of cause and effect.

Dr. Churchill says: "As in all cases where a disease is epidemic, it is and must ever be a difficult thing as to the contagiousness of puerperal fever; still I confess that the facts would lead one to the inference that, at least, it is communicable from a woman laboring under it to others in the same ward."

We cannot follow Dr. Churchill into a minute detail on the question of the conveyance of an imaginary contagion, by physicians. We shall examine a single point of this question, in order to show the character of the logic on which the hypothesis before us stands. Dr. Churchill says: "Dr. Gooch mentions that several instances occurred of peurperal fever attacking the patients of one practitioner, while those of others were exempt." Now occurrences precisely analogous to these have often happened in chills and fever, and by parity of reasoning we should be justified in thinking that disease infectious. Dr. Gooch continues, in reference to the facts quoted above: "One instance of this kind was very remarkable: a general practitioner in large midwifery practice lost so many patients from puerperal fever, that he determined to deliver no more for sometime, but that his partner should attend in his place. This plan was pursued for one month, during which not a case of the kind occurred in their practice. The elder practitioner, being then sufficiently recovered, returned to his practice, but the first patient he attended was attacked by the disease and died." It is on such data as these statements that the contagiousness of puerperal fever stands. We think with Dr. Churchill, that Dr. Gooch's facts "prove too much," and, therefore, they prove nothing. In addition to believing that "the clothes or person may preserve the contagion for a month," we are required to swallow the absurdity that the clothes or person of one individual may carry the contagion in a latent state, for a month, while another person in active contact with patients, could not acquire the contagious property in a month! This is at once absurd and ridiculous.

Dr. Copeland has gathered a great many statements for the purpose of proving the contagiousness of purperal fever. But we hope no one will be infected by Dr. Copeland. On the subjects of infection and contagion he seems to have a monomania, that is distressing to his readers.

We have thus brought down the condition of the literature of puerperal fever to the present period, and must now turn to other portions of Dr. Churchill's book. The labors of the editor will be indicated by the enumeration of the essays to be found in the work. The following are the titles of the various papers:

"Dr. Denman on the Puerperal Fever.

Dr. Hulme on Puerperal Fever.

Dr. Leake on the Childbed Fever.

Mr. Charles White on Pregnant and Lying-in Women.

Dr. Kirkland on Childbed Fevers.

Dr. Butler on the Puerperal Remittent Fever.

Dr. Joseph Clarke on the Puerperal Fever.

Dr. John Clarke on the Management of Pregnancy and Labor.

Dr. Gordon on the Epidemic Puerperal Fever.

MISCELLANEOUS ESSAYS.—Dr. Fothergill on the Management Proper at the Cessation of the Menses.

Dr. Macbride's Cases of Tumefaction of the Labium after Delivery.

Dr. Clarke on Cauliflower Excrescence from the Os Uteri.

Dr. Clarke's two Cases of Tumor of the Uterus.

Dr. Denman's Account of an Excrescence from the Womb."

We are thankful to the Sydenham Society for this excellent reprint of the monographs in this volume. They have rendered the science of medicine a very acceptable service, and not the least acceptable part of that service was in placing this undertaking in the hands of Dr.

Churchill. He has performed his duties in a way that reflects great credit upon him.

The American publishers have made a beautiful book of this. The typography is of a superior character, and everything about the mechanical part is tasteful and appropriate.

These various works may be found at the book-store of Beckwith & Morton.

